# Cross-scale energy cascade powered by magnetospheric convection

**DOI:** 10.1038/s41598-022-08038-x

**Published:** 2022-03-15

**Authors:** Aleksandr Y. Ukhorskiy, Kareem A. Sorathia, Viacheslav G. Merkin, Chris Crabtree, Alex C. Fletcher, David M. Malaspina, Steven J. Schwartz

**Affiliations:** 1 SES, Johns Hopkins University Applied Physics Laboratory, Laurel, 20723 USA; 2 Physics Department, Dartmouth College, Hanover, 03755 USA; 3 Plasma Physics Division, Naval Research Laboratory, Washington, DC 20375 USA; 4 Laboratory for Atmospheric and Space Physics, University of Colorado, Boulder, CO 80303 USA; 5 Astrophysical and Planetary Sciences Department, University of Colorado, Boulder, CO 80303 USA; 6 Emeritus, Blackett Laboratory, Imperial College London, London, SW7 2AZ UK

**Keywords:** Magnetospheric physics, Astrophysical plasmas

## Abstract

Plasma convection in the Earth’s magnetosphere from the distant magnetotail to the inner magnetosphere occurs largely in the form of mesoscale flows, i.e., discrete enhancements in the plasma flow with sharp dipolarizations of magnetic field. Recent spacecraft observations suggest that the dipolarization flows are associated with a wide range of kinetic processes such as kinetic Alfvén waves, whistler-mode waves, and nonlinear time-domain structures. In this paper we explore how mesoscale dipolarization flows produce suprathermal electron instabilities, thus providing free energy for the generation of the observed kinetic waves and structures. We employ three-dimensional test-particle simulations of electron dynamics one-way coupled to a global magnetospheric model. The simulations show rapid growth of interchanging regions of parallel and perpendicular electron temperature anisotropies distributed along the magnetic terrain formed around the dipolarization flows. Unencumbered in test-particle simulations, a rapid growth of velocity-space anisotropies in the collisionless magnetotail plasma is expected to be curbed by the generation of plasma waves. The results are compared with in situ observations of an isolated dipolarization flow at one of the Magnetospheric Multiscale Mission spacecraft. The observations show strong wave activity alternating between broad-band wave activity and whistler waves. With estimated spatial extent being similar to the characteristic size of the temperature anisotropy patches in our test-particle simulations, the observed bursts of the wave activity are likely to be produced by the parallel and perpendicular electron energy anisotropies driven by the dipolarization flow, as suggested by our modeling results.

## Introduction

Much of dynamics across Earth’s magnetosphere-ionosphere system are powered by a highly variable flow of the solar wind permeated by the interplanetary magnetic field (IMF). The solar wind-magnetosphere coupling is the strongest during intervals of the southward IMF, when magnetic reconnection at the subsolar magnetopause with a subsequent reconnection in the distant magnetotail sets off a global convection cycle of the magnetic flux from the dayside into the magnetotail and then back to the dayside magnetosphere. Known as the Dungey cycle^1^, it gives rise to a wide range of global processes including magnetospheric substorms^2^, the buildup of storm-time ring current^3^, field-aligned current generation^4^, and variability of radiation belt intensities^5^.

The textbook picture of magnetospheric convection invokes a large-scale duskward electric field, induced by the solar wind-magnetosphere coupling, which drives the Earthward bulk flow of cold plasma across the entire extent of the central plasma sheet^6^. As the plasma is convected towards the planet, into the regions of higher magnetic field intensity, it exhibits adiabatic energization in accordance with the conservation of the first two adiabatic invariants of the charged particle motion^7^. In a quasi-dipolar geomagnetic field the inward convection produces energization predominantly in the direction perpendicular to the ambient magnetic field^8^, which, as the particles reach the inner magnetosphere and are energized to 10-100 keV, provides the free energy for the electromagnetic ion cyclotron and whistler wave growth^9^.

The growing wealth of observational evidence and physics-based modeling over past two decades suggests that plasma convection from the distant magnetotail to the inner magnetosphere occurs largely in the form of mesoscale flows, i.e., discrete enhancements in the Earthward plasma flow localized to a few Earth radii in the azimuthal direction^10–12^. The mesoscale convection flows are typically preceded by sharp dipolarizations of magnetic field often referred to as dipolarization fronts or dipolarizing flux bundles^13,14^. To elucidate the fact that mesoscale flows are associated with localized dipolarizations of the ambient magnetic field, they are referred to as *dipolarization flows* hereafter. Dipolarization flows provide an effective mechanism of particle transport and acceleration^15,16^. During storms, mesoscale dipolarization flows produce ion and electron injections into the heart of the ring current, which can account for much of its energy density^17,18^.

Recent observational studies provided compelling evidence that the mesoscale convection may also be an energy source of a wide range of kinetic processes^19^ such as kinetic Alfvén waves^20^, whistler chorus waves^21^, nonlinear time-domain structures^22^, and shear flow-driven electron-ion hybrid modes at the leading edge of the flows^23,24^. In this paper we explore how the mesoscale convection produces velocity space instabilities in electron distributions, thus providing a pathway for the energy cascade from global to kinetic processes in Earth’s magnetotail. We employ test-particle simulations in our Conservative Hamiltonian Integrator of Magnetospheric Particles (CHIMP)^25^ one way coupled to a high-resolution magnetohydrodynamic (MHD) simulations of plasma convection in the magnetotail. For the latter we use the Lyon-Fedder-Mobarry (LFM) global magnetospheric model^26^. The next section describes results of the test-particle simulations and the subsequent analysis of the anisotropy formation process they reveal. In the “Discussion” section, followed by Conclusions, we describe and discuss the comparison of our simulation results with the observations of electron instabilities and wave activity measured at the Magnetospheric Multiscale spacecraft around an isolated dipolarization flow.

**Figure 1:**
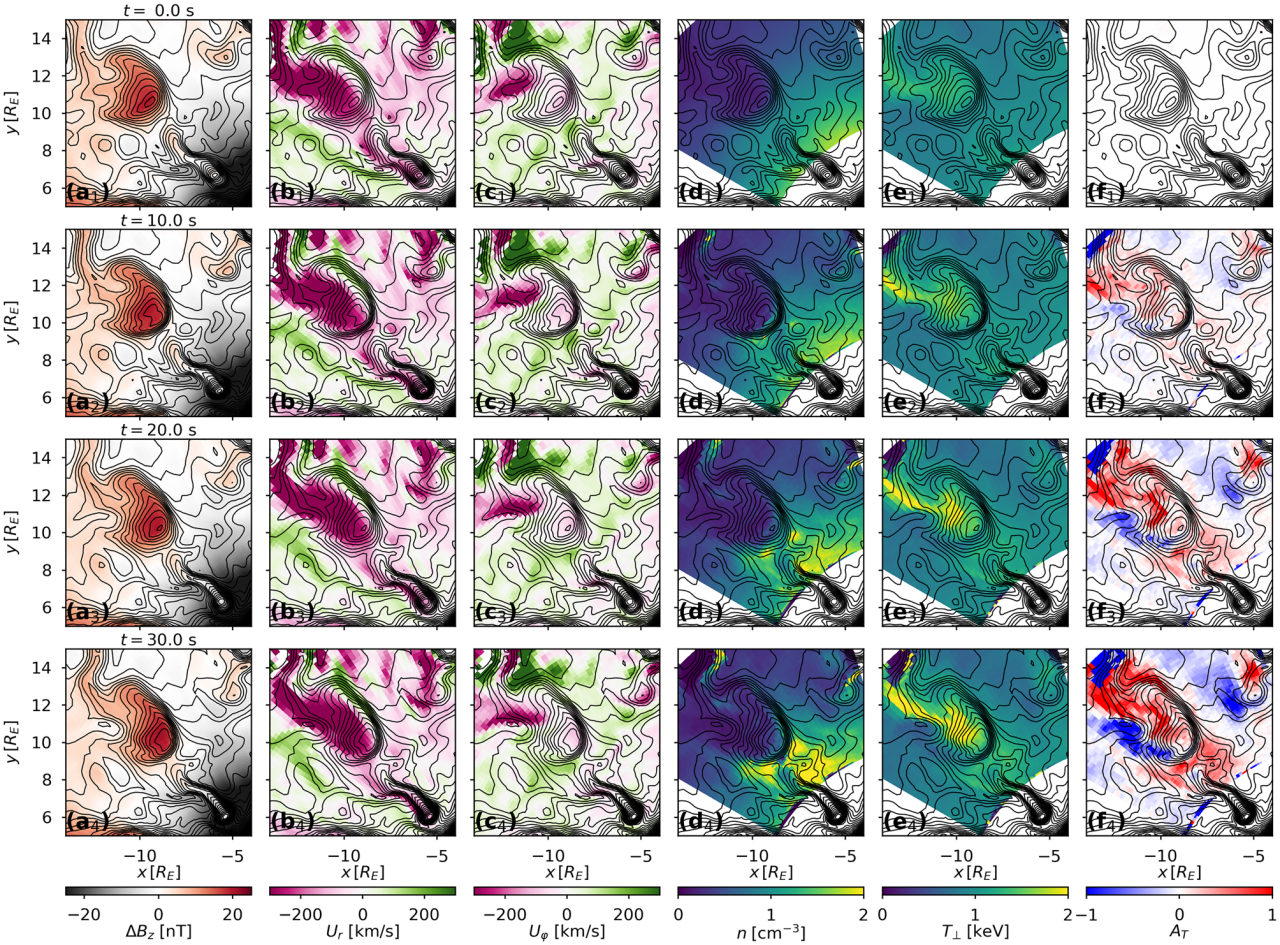
The growth of interchanging regions of parallel and perpendicular temperature anisotropies in electron distributions around a dipolarization flow in MHD and test-particle simulations. Electron PSD at $t=0$ were initialized with *T* and *n* from MHD simulations renormalized to a kappa distribution with $\kappa =3.5$ and $T_i/T_e=4$. Panels (**a**): external magnetic field, $\Delta B_z$; panels (**b**) and (**c**): the radial, $U_r$ and the azimuthal $U_\varphi$ components of the total equatorial drift velocity of 15 keV electrons; panels (**d**) and (**e**) the number density, *n*, and perpendicular temperature $T_\bot$ of electrons in the test-particle simulations; panels (**f**): electron temperature anisotropy, $A_T$. Negative (positive) values of $A_T$ correspond to temperature anisotropies parallel (perpendicular) to the ambient magnetic field direction. The contours of constant total magnetic field intensity are shown with black lines in all panels of the figure.

## Results

### Anisotropy formation at dipolarization front

According to our previous analysis^16,25^, complex *magnetic terrain*, carved by dipolarization flows in the ambient magnetic field plays a key role in the energization and transport of energetic particles from the magnetotail to the inner magnetosphere. In particular, magnetic islands formed around dipolarization fronts can stably trap particles enabling their inward transport by over 10 Earth radii, $R_e$, leading to increases in their energy by more than an order of magnitude.

To explore how electron dynamics in a dipolarization flow channel impact the shape of the electron distribution function we conduct test-particle simulations with our Conservative Hamiltonian Integrator of Magnetospheric Particles (CHIMP)^25^. A three dimensional test-particle simulation was conducted in the electromagnetic field computed in the Lyon-Fedder-Mobarry (LFM) high-resolution global MHD magnetospheric model^26^ during an interval of enhanced earthward convection^12^. At the simulation start, an ensemble of 40 million test-particles was initialized in an equatorial wedge centered at a large dipolarization front and distributed evenly over the entire range of the equatorial pitch angles from $0^\circ$ to $180^\circ$, and a wide energy range from 0.1 keV to 200 keV using a logarithmic spacing. Electron dynamics in the time varying electromagnetic field of the mesoscale convection were calculated in the guiding center approximation. Then, test-particle trajectories were used to compute the evolution of the electron phase space density under the assumption that initially particles had a kappa energy distribution and were fully isotropic in pitch angle (see “Test particle weighting” section of “Methods” for details).

The results are summarized in Fig. 1 showing simulation snapshots captured at a 10 second cadence along the magnetic equator. Figure 1a panels show the snapshots of the external magnetic field, $\Delta B_z$ (i.e., total magnetic field with Earth’s dipolar field subtracted from it). Figure 1b and Fig. 1c contain the radial, $U_r$, and the azimuthal, $U_\varphi$ components of the combined $E\times B$ and gradient-curvature drift computed for 15 keV electrons. Figure 1d and e depict the partial density, *n*, and perpendicular temperature, $T_\bot$, of electrons in our simulations, i.e., above the energy of 0.1 keV. Finally, Fig. 1f panels show evolution of the temperature anisotropy, $A_T$, of the electron distribution computed as:

1 $$\begin{aligned} A_T=\frac{T_\bot }{T_\Vert }-1,\; T_\bot \ge T_{\Vert };\; \mathrm{or}\;A_T=-\frac{T_\Vert }{T_\bot }+1,\; T_\bot < T_{\Vert }, \end{aligned}$$

where $T_\bot$ and $T_\Vert$ are electron temperatures perpendicular and parallel to the magnetic field.

In all panels of Fig. 1 contours of constant total magnetic field are shown with black lines. Closed contours indicate the magnetic islands associated with the dipolarization flows that enable stable trapping of energetic particles produced by a sharp gradient in the magnetic field at the interface of the dipolarization fronts embedded in the flows. As is shown in Fig. 1f panels, dipolarization flows also lead to rapid emergence of interchanging regions of parallel and perpendicular temperature anisotropies. It took less that 20 seconds for the anisotropy values to grow above $|A_T|=1$ out of the initially isotropic pitch-angle distribution. While being a test-particle model CHIMP does not account for particle feedback onto the fields, it is reasonable to anticipate that in the real-world plasma such rapid surge of temperature anisotropies would quickly make electron distributions unstable to wave growth, thus providing the pathway for the energy cascade from mesoscale to kinetic structures.

**Figure 2:**
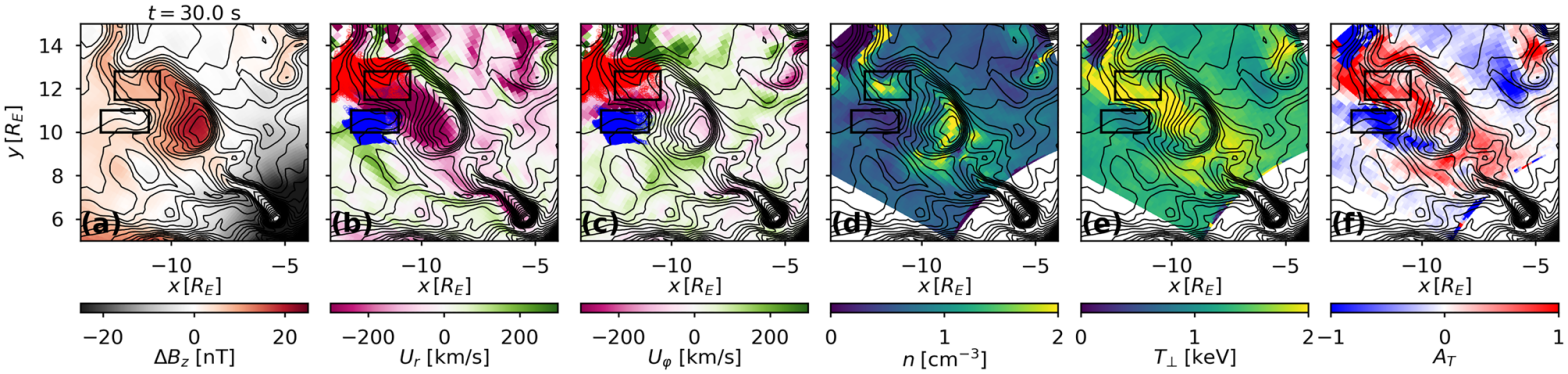
The snapshot of temperature anisotropies in the test-simulations (same as used in obtaining the results shown in Fig. 1), re-initialized with an isotropic kappa distribution with $$\kappa =3.5$$, $$T_e=1$$ keV, and $$n_{TP}=1 $$ $$\mathrm cm^{-3}$$, at the end of a 30 s simulation. Panels (**b**) and (**c**) show equatorial projections of test particle trajectories traced backward in time out of two regions of parallel (blue) and perpendicular (red) electron temperature anisotropies outlined with back rectangles in all the panels.

### Path to anisotropies

Since the initial set up of the test-particle simulations shown in Fig. 1 was derived from the MHD state with pronounced mesoscale variations in both *n* and *T*, the electron distribution, $$f(\mathbf{r}, \alpha , K)$$, started off with a builtin mesoscale structure both in $$\mathbf{r}$$ and in *K*, where $$\mathbf{r}$$ is the location in the equatorial plane, $$\alpha $$ is the equatorial value of the pitch angle, and *K* is the kinetic energy. To isolate the process underlying the emergence of temperature anisotropies, we removed any builtin mesoscale structuring by re-initializing previous test-particle simulation with a kappa distribution function isotropic in pitch angle and with $$\kappa =3.5$$, $$T=1$$ keV, and $$n=1 $$ $$\mathrm cm^{-3}$$, kept constant everywhere in the simulation domain. The results at the end of a 30 second simulation with the new phase space density initial conditions are summarized in Fig. 2 in the format similar to Fig. 1. As is evident from Fig. 2f, it exhibits strong pitch-angle anisotropies, with the pattern and the values similar to Fig. 1f$$_4$$.

**Figure 3:**
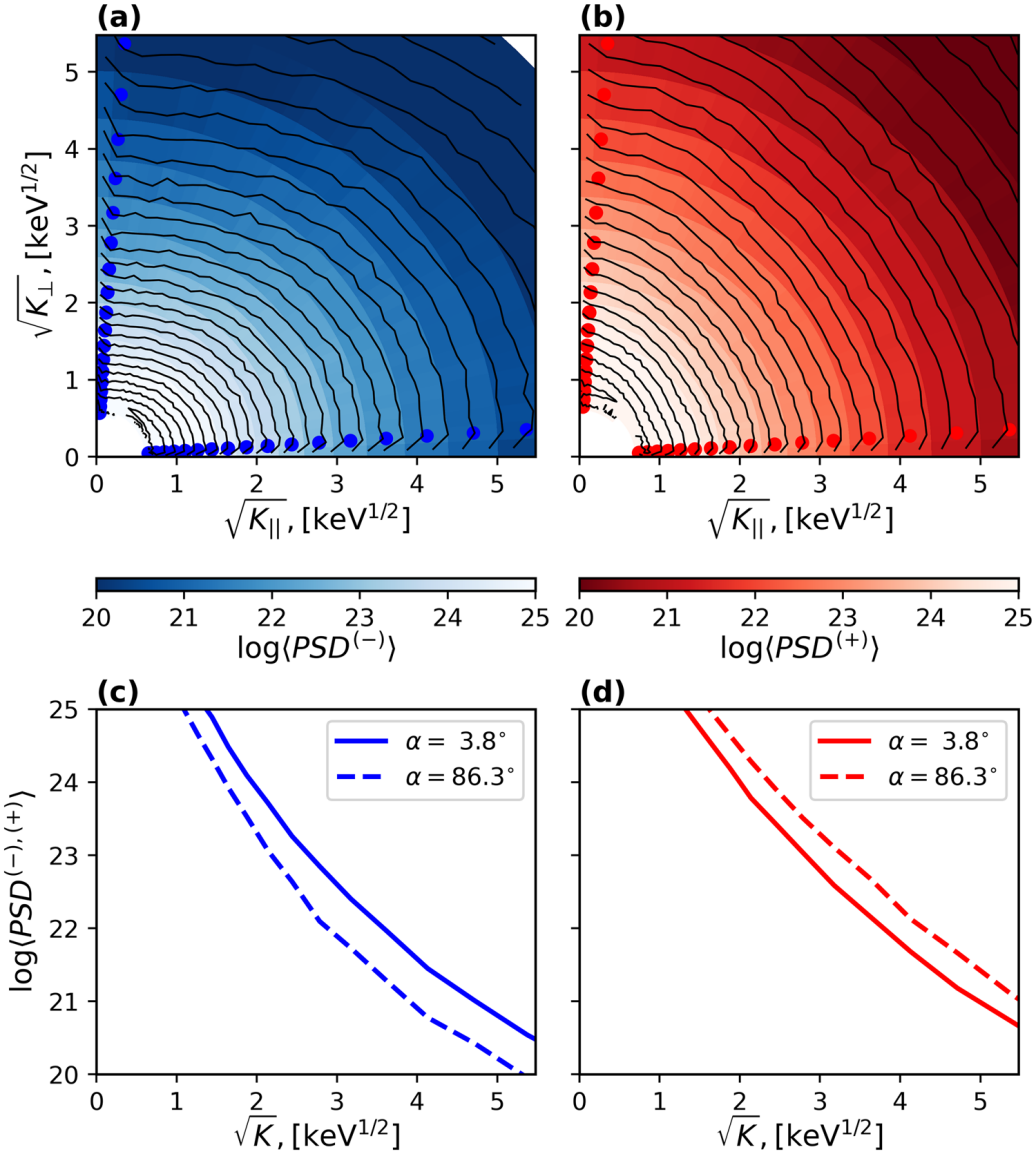
The parallel (panels (**a**) and (**c**)) and perpendicular (right panels (**b**) and (**d**)) anisotropies in electron distributions around dipolarization flows have similar values over the entire electron energy range. Panels (**a**) and (**b**) show electron PSD, averaged over the regions of parallel and perpendicular anisotropies shown in Fig. 2. The line plots in panels (**c**) and (**d**) show cuts of electron PSD at small and large pitch-angle values (indicated with symbols in panels (**a**) and (**b**)).

For further analysis, we used the anisotropy distribution at the end of the simulation interval (Fig. 2f) to select two regions with pronounced values of the parallel and perpendicular temperature anisotropies (marked with black rectangular outlines). Figure 3 shows details of the electron phase space density distributions averaged over the parallel (left panels) and the perpendicular (right panels) anisotropy regions. From the contour plots (top panels), and the line plots of the energy dependence of the electron distributions at small and large pitch-angle values (bottom panels), it follows that the anisotropies were produced by a process independent of electron energies, i.e., the anisotropy levels are similar over the entire energy range of the electron distribution. Considering that the perpendicular anisotropy emerged inside the inward dipolarization flow, whereas the parallel anisotropy formed outside of the dipolarization channel in the outward back flow, it is reasonable to assume that the anisotropies were attributed to the betatron effect.

To verify whether the emergence of interchanging patches of the parallel and perpendicular anisotropies is indeed controlled by the betatron effect, we selected large samples of test-particles from the final state of the simulations in the two selected anisotropy regions in Fig. 2f and followed their trajectories backward in time. To avoid tainting our consideration by energy dispersion in the gradient-curvature drift we considered electrons with the initial energy between 10 and 15 keV, which provide the largest contribution to temperature anisotropies, computed from the maximum absolute value of the partial anisotropy: $\Delta A_T(K)=\frac{\partial A_T}{\partial K}\Delta K$. Distributions of the equatorial projections of the test particles from both ensembles are shown in Fig. 2b and c with blue and red. As can be seen from the figure, the trajectories contributing to sculpting perpendicular and parallel temperature anisotropies, were separated by sharp features of the magnetic terrain around the dipolarization flow and hence did not overlap. The parallel anisotropy is formed by electrons drifting outward into the region of depleted magnetic field, whereas the perpendicular anisotropy is produced by electrons flowing inward towards higher magnetic field intensity.

**Figure 4:**
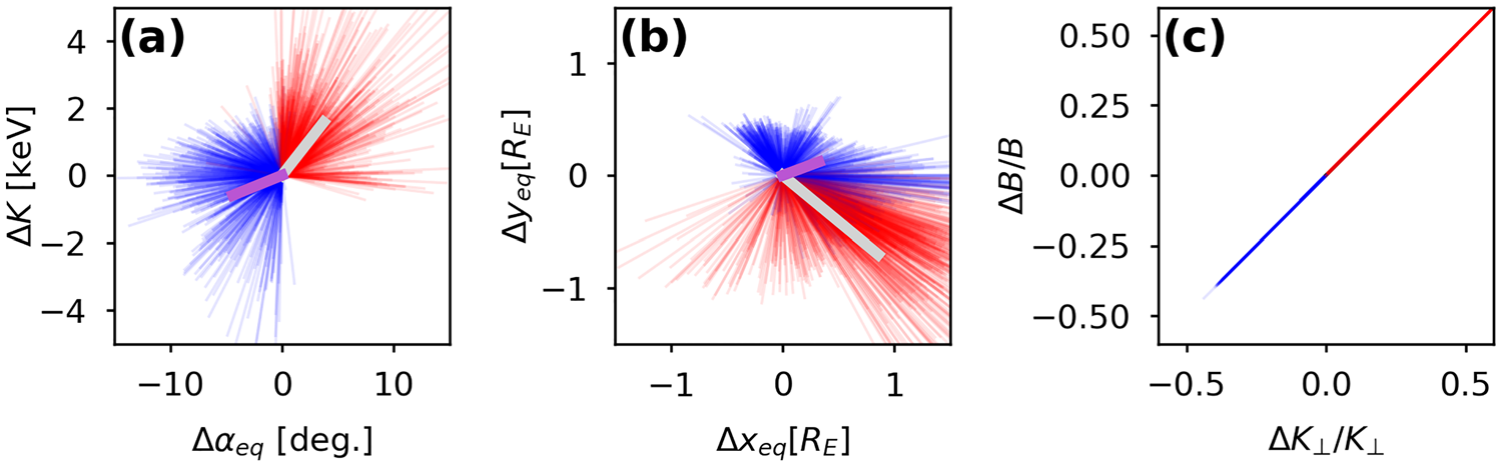
Over the course of the simulations, electron populations from the regions of parallel and perpendicular anisotropies, shown in Fig. 2, do not overlap and undergo changes that point to the betatron affect as the mechanism of their generation. The figure shows the changes in the electron energy and pitch-angle (panel (**a**), equatorial location (panel (**b**)), and the equatorial values of the electron perpendicular energy and total magnetic field intensity at electron location (panel (**c**)) at the end of the 30 s simulation interval for particles in the regions of parallel (blue color) and perpendicular (red color) anisotropies.

Figure 4 shows the difference between the initial and the final states in the particle ensembles contributing to the parallel (blue color) and the perpendicular (red color) anisotropies in the electron distribution. Parallel anisotropy was created by particles that (on average) lost their energy and decreased the equatorial pitch angle, whereas the perpendicular anisotropy was formed by particles that were (on average) energized and increased the pitch angle (Fig. 4a). Figure 4b, which is showing the change in the particle equatorial location, well illustrates the absence of mixing between the two ensembles. Finally, the direct proportionality between the variations of the electron perpendicular energy and the changes in the magnetic field intensity in their locations, shown Fig. 4c, confirms that the anisotropies were indeed formed due to the betatron effect.

**Figure 5:**
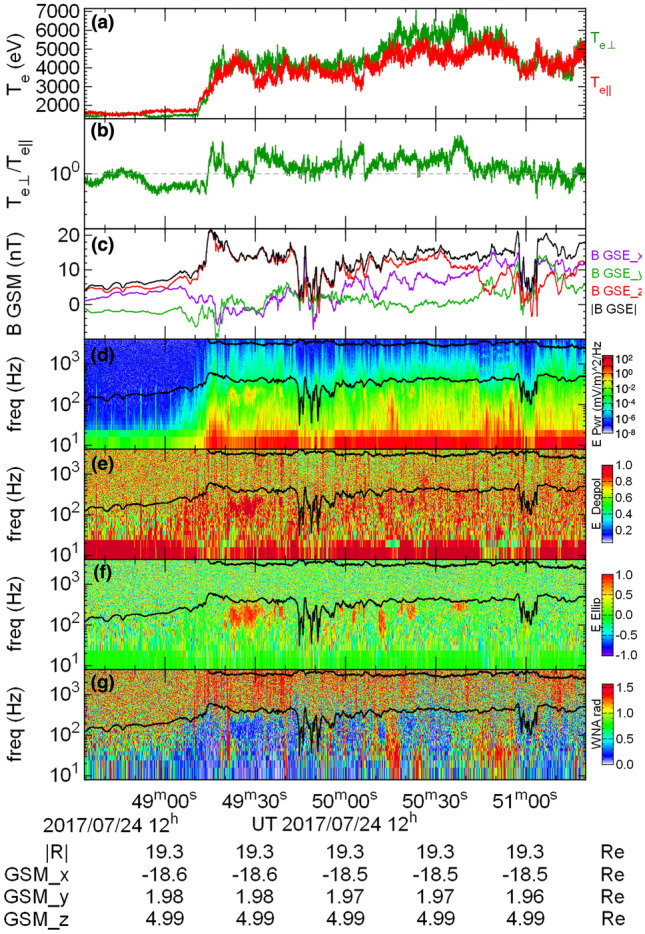
In situ observations of the wave activity and electron distribution in a dipolarization flow at MMS3 are fully consistent with the emergence of the mesoscale pattern of interchanging regions of parallel and perpendicular anisotropies predicted in our test particle simulations. Panel (**a**): electron temperature; panel (**b**): the ratio of the perpendicular and parallel temperatures - the scale is equivalent to $-1< A_t < 1$ used in Fig. 1; panel (**c**): magnetic field intensity; panel (**d**): electric field power spectral density; panel (**e**) electric field polarization; panel (**f**): electric field ellipticity, with negative (positive) values indicating left (right) hand polarization; panel (**g**): wave normal angle. Black traces in (**d**)–(**g**) are the electron plasma frequency (upper trace) and electron cyclotron frequencies (lower).

## Discussion

To examine whether the emergence of mesoscale patches of perpendicular and parallel anisotropies in the electron distribution function at dipolarization flows, as predicted by our test-particle simulations, does occur in Earth’s magnetotail we use *in situ* observations from the Magnetospheric Multiscale (MMS) mission^27^: the magnetic field from the Fluxgate Magnetometer^28^, plasma wave activity measured by the electric field probes^29,30^, and the electron data from Fast Plasma Investigation^31^ (for details see “MMS data analysis” section of “Methods”). Figure 5 shows MMS3 observations of a dipolarization flow on 07/24/2017 at approximately 19.3 $R_E$ in the magnetotail close to the midnight meridian. The dipolarization event was registered at approximately 12:49:30 UT, as is evident from a sharp increase in the magnetic field $B_z$ component (Fig. 5c) along with an enhancement in the electron temperature (Fig. 5a).

From the ratio of the electron perpendicular and parallel temperatures shown in Fig. 5b it follows that the electron distribution exhibited a parallel anisotropy ahead of the dipolarization flow front and a weak perpendicular anisotropy inside the flow. However, considering that any excess of free energy attributed to temperature anisotropies is expected to be quickly removed via generation of plasma waves, for further insight into the instabilities driven by the flow it is necessary to examine the wave activity associated with the event (Fig. 5d–g). Power spectrogram of the electric field data is shown in Fig. 5d. Figure 5e shows the degree of polarization (when it is near unity (zero), the polarization is circular (linear)). Wave ellipticity and wave normal angle are shown in Figs. 5f and g. As can be seen from the figure, the wave activity inside the dipolarization flow was comprised of intermittent bursts of whistler waves between approximately 100 and 400 Hz, propagating near-parallel to the ambient magnetic field, and broad-band wave activity, extending to above 1 kHz. With a typical flow speed of several hundred km/s, the spatial extent of the observed wave bursts is expected to be of the order of $\lesssim 1$ $R_E$, similar to the size of the anisotropy patches in our test-particle simulations.

The presence of intermittent bursts of whistler and broad-band wave activity observed in the dipolarization flow is fully consistent with a rapid growth of a mesoscale pattern of interchanging regions of the electron perpendicular and parallel temperature anisotropies as predicted in the test-particle simulations. Perpendicular temperature anisotropies are expected to produce parallel whistler mode instabilities^9^. Since the whistler instability is typically convective and the whistler group velocity is large, a remnant temperature anisotropy can remain alongside with the waves, as was the case shown in Fig. 5b. The regions of parallel anisotropy can be a source to the firehose instability that can operate on time-scales of the order of the electron cyclotron frequency^32^ and produce the observed broad-band wave activity. Because the firehose instability is absolute and the modes it generates are non-propagating it is expected for the temperature anisotropy to be quickly relaxed, which would explain the absence of $T_\Vert >T_\bot$ inside the dipolarization flow in Fig. 5b. Similarly, in the solar-wind the electron firehose instability is often used to explain the lack of observations with parallel temperature anisotropies. Two-dimensional simulations of the electron-firehose instability showed the temperature anisotropy quickly relaxed and left behind remnant whistler mode waves^33^.

## Conclusions

In this study we used our one-way coupled global MHD and test-particle simulations to explore the growth of velocity-space anisotropies at Earthward propagating dipolarization flows, and then compared the simulation results with the *in situ* wave and plasma observations of an isolated dipolarization flow at the MMS3 spacecraft. Our conclusions, built on the earlier results^16^, further contribute to the paradigm shift in the magnetospheric convection as a complex cascade of coupled processes from global plasma instabilities and magnetic field reconfiguration, to mesoscale flows and dipolarization fronts, that power kinetic instabilities and wave structures:

- Earthward plasma convection in the magnetotail largely consists of mesoscale flows that exhibit complex magnetic terrain with features such as magnetic islands, which are instrumental to inward transport and energization of suprathermal particles.
- Mesoscale flows produce interchanging regions of parallel and perpendicular temperature anisotropies by polarizing electron distributions along the magnetic terrain boundaries with subsequent adiabatic heating and cooling (betatron effect).
- Rapid growth of mesoscale patches of the parallel and perpendicular temperature anisotropies in the electron distributions around dipolarization flows can provide free energy for the generation of broad-band and whistler waves reported from spacecraft observations.

The most intriguing of the above results, unanticipated from the textbook picture of magnetospheric convection, is the fact that when broken down into mesoscale flows, the convection produces not only strong temperature anisotropies perpendicular to the ambient magnetic field, but also equally pronounced anisotropies parallel to the ambient field. With the surprisingly rapid growth rates of the order of only a few electron bounce periods, these anisotropies are likely to be a source of free energy for the generation of wide range of plasma waves and structures observed in association with dipolarization flows.

## Methods

### Test particle weighting

To track global evolution of the particle phase space density (in this case electrons) we use a four-dimensional hyperplane corresponding to the magnetic equator: $\mathbf{X}=(r,\varphi ,K,\alpha )$ with a volume element of $\delta \Gamma (\mathbf{X})=\delta p\delta V(r,\varphi ,\alpha )$, where *r* and $\varphi$ are the distance from the Earth’s center and the azimuthal angle in the hyper plane of the magnetic equator defined by the magnetic field minima along the magnetic field lines, *K* is the particle kinetic energy, $\alpha$ is the equatorial pitch angle, *p* is the momentum, and $\delta V$ is the is the flux tube volume attached to the equatorial surface area of $rdrd \varphi$, which is accessible to a given pitch-angle particle:

2 $$\begin{aligned} \delta V=B(r,\varphi )rdrd \varphi \int _{m}^{m^\prime }\frac{dl}{B} \end{aligned}$$

where the integration on the right side is carried between the conjugate bounce points of a particle with the pitch angle $\alpha$ above and below the magnetic equator.

The gyrophase-integrated momentum space volume element is given by:

3 $$\begin{aligned} \delta p = 2\pi \sin \alpha d\alpha \frac{(mc^2)^2}{c^3}\sqrt{\gamma ^2-1}\gamma dK\simeq 2\pi \sin \alpha d\alpha \sqrt{2}\frac{(mc^2)^{3/2}}{c^3}\sqrt{K}dK, \end{aligned}$$

where the approximate equality on the right hand side holds in a non-relativistic limit, *c* is the speed of light, $\gamma$ is the relativistic factor, and *m*.

The phase space density $f(\mathbf{X})$ is then computed from $\delta N(r,\varphi ,K,\alpha )=f(r,\varphi ,K,\alpha )\delta p \delta V$, the number of all particles with the equatorial pitch angle $\alpha$ and the energy *K* in the flux tube with the $rdrd\varphi$ area centered at the $(r,\varphi )$ point of the magnetic equator. The phase space coordinates, $(r,\varphi ,K,\alpha )$, of a particle at the point $\mathbf{r}=(x,y,x)$ off the equatorial plane with the pitch angle $\alpha ^\prime$ and the energy *K* are computed from the instantaneous field-aligned projection under the conservation of energy and the first adiabatic invariant: $K=\mathrm{const}$, $\sin ^2\alpha =\sin ^2\alpha ^\prime B(r,\varphi )/B(\mathbf{r})$.

To initialize the electron phase space density we assume that at the simulation start the plasmasheet electron distribution is isotropic in pitch-angle and has a kappa distribution in energy^34^:

4 $$\begin{aligned} f(r,\varphi ,K,\alpha )=\frac{4\pi n_0}{(2\pi m_e\kappa K_0)^{3/2}}\frac{\Gamma (\kappa +1)}{\Gamma (\kappa -1/2)}\left( 1+\frac{K}{\kappa K_0}\right) ^{-(1+\kappa )}, \end{aligned}$$

with $K_0 = T_0(1-3/2\kappa )$, the plasma density $n_0 = n_{MHD}(r,\varphi ,t=0)$ and temperature $T_0 = \frac{1}{4}T_{MHD}(r,\varphi ,t=0)$ given by the global MHD simulations, and $\kappa = 3.5$ in accordance with statistical properties of the plasmasheet dipolarization flows at 15 $R_E$^35^.

At the simulation start, $t=0$, each test particle in a given cell of the phase space grid, $\{\mathbf{X}^n\}$, is assigned with a weight according to the ratio of the number of the “real” particles, $f\delta \Gamma$, to the number of test-particles, $N_m$, in this grid cell:

5 $$\begin{aligned} \begin{aligned}{}&w_m = \frac{1}{N_m}f(t=0,\mathbf{X}^n)\delta \Gamma (\mathbf{X}^n ),\;N_m=\#{\mathscr {I}} \\&{\mathscr {I}}=\{\mathbf{X}_m(t=0):{\mathbf{X}}_m\in \left[ {\mathbf{X}}^n,\mathbf{X}^n+\delta \mathbf{X}^n\right) \} \end{aligned} \end{aligned}$$

Global evolution of the electron phase space density at $t>0$ is then computed from particle weights as:

6 $$\begin{aligned} \begin{aligned}{}&f(t>0,\mathbf{X}^n)=\frac{1}{\delta \Gamma (\mathbf{X}^n)}W_\sigma (\mathbf{X}^n);\;W_\sigma (\mathbf{X}^n)=\sum _{m=1}^{N_m} w_m,\;N_m=\#\mathscr {J}\\&\mathscr {J}=\{\mathbf{X}_m(t>0):\mathbf{X}_m\in \left[ \mathbf{X}^n,\mathbf{X}^n+\delta \mathbf{X}^n\right) \} \end{aligned} \end{aligned}$$

For test-particle weighting and phase space density calculations in this study we sued a regular 81 × 44 × 30 × 37 grid in $(r,\varphi ,K,\alpha )$ with logarithmic spacing in *K*. With $4\cdot 10^7$ test-particles, used in the simulation, it corresponds to approximately 10 test particles in each cell of the phase space grid and as many as approximately $10^4$ test particles in the configurational $(r,\varphi )$ space of the phase space density moments *n*, *T*, *A*, which insured low noise levels.

### MMS data analysis

The MMS electron data used in this study and shown in Fig.  5 are drawn from the level 2 science moments provided by the instrument team^31^ and held at the mission science data center (see the “Acknowledgements” section). The temperature moments are the direct integration of the full 3D velocity space sampled at 32 energies, 16 elevation and 32 polar angles every 30ms in burst mode for this interval. The moment algorithms remove or correct for the influence of the spacecraft potential, and the presence of photoelectrons and internally produced secondary electrons.

The electric field data used to diagnose plasma waves in this study (Fig. 5) are drawn from the level 2 science data provided by the FIELDS instrument team^36^ and held at the mission science data center (see the “Acknowledgements” section). To derive the power spectra and cross-spectral analysis results shown in Fig. 5 the 8,192 sample per second 3-axis time series electric field data are first rotated into magnetic field-aligned coordinates. The DC-coupled magnetic field data used to perform this rotation are the four-spacecraft barycentric magnetic field data, sampled at 16 Hz. A windowed Fast Fourier Transform (FFT) is then applied to the magnetic field-aligned electric field data. The FFT used has 2048 points, 50% overlap between windows, and a Hanning window applied.

From the real and imaginary FFT outputs, A 3×3 cross-spectral matrix is determined for each electric field spectrogram time and frequency bin shown in Fig. 5. The power spectral matrix shown is the sum of the squares of the cross-spectral matrix diagonal elements. The cross-spectral matrix data are smoothed in time with a Hanning-based smoothing profile for the remaining cross-spectral analysis.

Wave properties are defined from the cross-spectral matrices using standard definitions. The wave normal angle is defined as the angle between the magnetic field direction and the minimum variance direction of the 3D wave field for a given time and frequency bin^37^. Ellipticity is defined as the ratio of the smallest to largest eigen value of the cross-spectra matrix defined in the 2D plane transverse to the magnetic field direction. The sign indicates the handedness (+ for right-handed, − for left-handed) of the rotation of the electric field vector in this plane^37^. Degree of polarization (Fig. 5e) is defined as in^38^. When degree of polarization is near one, the wave polarization is close to circular. When it is near zero, the wave polarization is close to linear.
